# Supplementary material for: Genetic Relationships and Signatures of Adaptation to the Climatic Conditions in Populations of Apis cerana Based on the Polymorphism of the Gene Vitellogenin
Source: Insects. 2022 Nov 15;13(11):1053. doi: 10.3390/insects13111053 (PMC9694869; doi:10.3390/insects13111053)
Supplement: Supplementary file 1 [file insects-13-01053-s001.zip › Supplementary material.pdf]

Supplementary data

Table S1. The accession numbers of the gene *VG* exons of *A. cerana* honey bees submitted to the GenBank

No.	Samples	GenBank accession numbers of the gene *VG* exons (2-7)
1.	*A.*_*cerana*,_Gangneung,_Korea_01	MH755718, MH755753, MH755788, MH755823, MH755858, MH755893
2.	*A.*_*cerana*,_Gangneung,_Korea_02	MH755719, MH755754, MH755789, MH755824, MH755859, MH755894
3.	*A.*_*cerana*,_Gangneung,_Korea_03	MH755720, MH755755, MH755790, MH755825, MH755860, MH755895
4.	*A.*_*cerana*,_Goseong,_Korea_01	MH755733, MH755768, MH755803, MH755838, MH755873, MH755908
5.	*A.*_*cerana*,_Hiroshima,_Japan_01	MH755739, MH755774, MH755809, MH755844, MH755879, MH755914
6.	*A.*_*cerana*,_Hiroshima,_Japan_02	MH755740, MH755775, MH755810, MH755845, MH755880, MH755915
7.	*A.*_*cerana*,_Hongcheon,_Korea_01	MH755717, MH755752, MH755787, MH755822, MH755857, MH755892
8.	*A.*_*cerana*,_Icheon,_Korea_01	MH755714, MH755749, MH755784, MH755819, MH755854, MH755889
9.	*A.*_*cerana*,_Icheon,_Korea_02	MH755715, MH755750, MH755785, MH755820, MH755855, MH755890
10.	*A.*_*cerana*,_Icheon,_Korea_03	MH755716, MH755751, MH755786, MH755821, MH755856, MH755891
11.	*A.*_*cerana*,_Kathmandu,_Nepal_01	MH755743, MH755778, MH755813, MH755848, MH755883, MH755918
12.	*A.*_*cerana*,_Kathmandu,_Nepal_02	MH755744, MH755779, MH755814, MH755849, MH755884, MH755919
13.	*A.*_*cerana*,_Kitahiroshima,_Japan_01	MH755741, MH755776, MH755811, MH755846, MH755881, MH755916
14.	*A.*_*cerana*,_Kitahiroshima,_Japan_02	MH755742, MH755777, MH755812, MH755847, MH755882, MH755917
15.	*A.*_*cerana*,_Okcheon,_Korea_01	MH755723, MH755758, MH755793, MH755828, MH755863, MH755898
16.	*A.*_*cerana*,_Okcheon,_Korea_02	MH755724, MH755759, MH755794, MH755829, MH755864, MH755899
17.	*A.*_*cerana*,_Okcheon,_Korea_03	MH755725, MH755760, MH755795, MH755830, MH755865, MH755900
18.	*A.*_*cerana*,_Samcheok,_Korea_01	MH755721, MH755756, MH755791, MH755826, MH755861, MH755896
19.	*A.*_*cerana*,_Samcheok,_Korea_02	MH755722, MH755757, MH755792, MH755827, MH755862, MH755897
20.	*A.*_*cerana*,_Sancheong,_Korea_01	MH755734, MH755769, MH755804, MH755839, MH755874, MH755909
21.	*A.*_*cerana*,_Sancheong,_Korea_02	MH755735, MH755770, MH755805, MH755840, MH755875, MH755910
22.	*A.*_*cerana*,_Sancheong,_Korea_03	MH755736, MH755771, MH755806, MH755841, MH755876, MH755911

23.	*A.*_*cerana*,_Sangju,_Korea_01	MH755726, MH755761, MH755796, MH755831, MH755866, MH755901
24.	*A.*_*cerana*,_Sangju,_Korea_02	MH755727, MH755762, MH755797, MH755832, MH755867, MH755902
25.	*A.*_*cerana*,_Sangju,_Korea_03	MH755728, MH755763, MH755798, MH755833, MH755868, MH755903
26.	*A.*_*cerana*,_Taiwan,_China_01	MH755747, MH755782, MH755817, MH755852, MH755887, MH755922
27.	*A.*_*cerana*,_Taiwan,_China_02	MH755748, MH755783, MH755818, MH755853, MH755888, MH755923
28.	*A.*_*cerana*,_Uisung,_Korea_01	MH755729, MH755764, MH755799, MH755834, MH755869, MH755904
29.	*A.*_*cerana*,_Uisung,_Korea_02	MH755730, MH755765, MH755800, MH755835, MH755870, MH755905
30.	*A.*_*cerana*,_Uisung,_Korea_03	MH755731, MH755766, MH755801, MH755836, MH755871, MH755906
31.	*A.*_*cerana*,_Vladivostok,_Russia_01	MH755745, MH755780, MH755815, MH755850, MH755885, MH755920
32.	*A.*_*cerana*,_Vladivostok,_Russia_02	MH755746, MH755781, MH755816, MH755851, MH755886, MH755921
33.	*A.*_*cerana*,_Wanju,_Korea_01	MH755732, MH755767, MH755802, MH755837, MH755872, MH755907
34.	*A.*_*mellifera*,_Tamil_Nadu,_India_01	MH755737, MH755772, MH755807, MH755842, MH755877, MH755912
35.	*A.*_*mellifera*,_Tamil_Nadu,_India_02	MH755738, MH755773, MH755808, MH755843, MH755878, MH755913
Sequences of the gene *VG* exons (2-7) retrieved from the GenBank		
36	*A.*_*cerana*,_ACSNU-2.0_Seoul,_Korea_01	ACSNU-2.0, PRJNA235974, NW_016017501 compl(417059..423214)
37	*A.*_*cerana*,_*Apis*CC1.0_Jiangxi,_China_01	ApisCC1.0, PRJNA239323, KZ288206 (781683-788045)
38	*A.*_*cerana*,_*Apis*cer_1.0_Kyoto,_Japan_01	Apiscer_1.0, PRJDB5799, BDUG01002346 (114156-120605)
39	*A.*_*cerana*,_KP119837_Yunnan,_China_01	KP119837
40	*A.*_*cerana*,_KP398512_Beijing,_China_01	KP398512
41	*A.*_*cerana*,_KT725235_Yunnan,_China_02	KT725235
42	*A.*_*cerana*,_NM_001328484_Beijing,_China_02	NM_001328484
43	*A.*_*mellifera*,_M2161,_Warsaw,_Poland_01	JN557285, JN557377, JN557191, JN557563, JN557471, JN557099

Table S2. The number of nucleotide differences (transitions + transversions) between *A. cerana* local populations and *A. mellifera* outgroups based on exon sequences of the gene *VG* below the diagonal and its standard error (S.E.) above the diagonal

		**1**	**2**	**3**	**4**	**5**	**6**	**7**	**8**	**9**	**10**	**11**	12	13	14	15	16	17	18	19	20	21
		Standard error S.E.																				
**1***	Number of nucleotide differences (tr + tv)		**3.4**	**3.6**	**2.7**	**2.7**	**3.0**	**4.0**	**3.0**	**3.4**	**3.8**	**3.2**	2.9	3.9	4.8	4.7	4.3	4.6	4.5	4.6	5.6	6.6
**2**		**17**		**3.4**	**3.5**	**3.1**	**4.3**	**4.9**	**3.3**	**2.4**	**2.5**	**4.2**	3.5	3.5	4.2	4.9	5.2	4.2	4.2	3.8	6.9	6.7
**3**		**17**	**10**		**3.9**	**3.4**	**4.7**	**5.1**	**3.5**	**3.0**	**2.6**	**4.8**	4.1	3.7	4.8	5.3	5.5	4.8	5.0	4.8	6.9	7.3
**4**		**12**	**14**	**13**		**2.4**	**3.3**	**4.9**	**3.1**	**2.6**	**3.7**	**4.1**	3.0	3.3	4.8	4.7	4.9	4.5	4.3	4.0	5.9	6.8
**5**		**13**	**13**	**16**	**12**		**2.8**	**3.6**	**3.3**	**2.6**	**3.0**	**3.2**	3.0	3.1	3.8	4.3	4.1	4.2	3.7	4.3	5.5	6.7
**6**		**15**	**21**	**20**	**15**	**15**		**3.1**	**3.5**	**3.8**	**4.0**	**4.0**	3.2	4.2	4.2	4.8	4.8	4.7	4.6	5.1	5.7	6.8
**7**		**17**	**24**	**26**	**19**	**16**	**16**		**4.2**	**4.6**	**4.8**	**4.1**	4.6	4.1	5.7	4.7	4.2	4.6	4.4	5.4	5.5	6.9
**8**		**16**	**15**	**17**	**17**	**16**	**20**	**21**		**3.5**	**3.3**	**4.1**	3.1	3.8	4.4	4.7	4.7	4.7	4.5	4.7	6.4	7.2
**9**		**18**	**11**	**13**	**12**	**14**	**18**	**27**	**20**		**2.7**	**4.2**	4.1	3.1	3.9	4.4	4.3	4.1	3.7	4.1	6.3	6.3
**10**		**21**	**12**	**13**	**18**	**17**	**22**	**28**	**20**	**15**		**4.6**	3.8	3.8	3.1	4.7	5.0	4.6	4.1	4.5	6.8	7.1
**11**		**14**	**20**	**24**	**16**	**14**	**18**	**13**	**20**	**20**	**24**		4.3	4.5	6.1	4.6	4.6	4.8	4.3	4.9	4.8	6.8
12		16	16	17	15	16	19	23	19	19	27	21		4.4	4.7	5.4	5.5	4.9	4.7	4.8	5.9	7.4
13		22	22	22	20	20	24	25	27	19	25	22	24		4.1	4.8	4.5	4.3	4.1	4.2	6.6	6.3
14		26	20	18	23	22	19	34	26	18	16	32	25	23		4.9	5.2	5.2	4.8	5.4	7.9	6.7
15		40	36	39	36	35	38	37	42	33	37	38	43	38	39		4.4	4.2	3.4	4.4	6.5	6.9
16		28	27	29	26	24	28	25	32	22	29	27	32	26	29	36		4.7	4.2	5.0	6.3	5.2
17		35	32	32	30	32	35	33	39	29	34	33	35	33	39	36	36		3.7	4.2	6.1	6.9
18		29	24	27	26	25	26	26	31	22	27	27	32	28	29	27	29	29		4.5	6.6	6.1
19		29	22	27	27	28	31	32	33	24	30	30	29	27	32	34	33	30	28		7.3	6.9
20		36	46	45	40	38	38	32	44	43	49	33	40	40	52	61	49	55	50	52		7.9
21		259	253	255	252	254	258	254	269	258	256	256	260	253	258	260	252	258	249	261	270	

Notes: 1. *A. cerana*, Gangneung, Korea, 2. *A. cerana*, Goseong, Korea, 3. *A. cerana*, Hongcheon, Korea, 4. *A. cerana*, Samcheok, Korea, 5. *A. cerana*, Sancheong, Korea, 6. *A. cerana*, Sangju, Korea, 7. *A. cerana*, Seoul, Korea, 8. *A. cerana*, Icheon, Korea, 9. *A. cerana*, Okcheon, Korea, 10. *A. cerana*, Uisung, Korea, 11. *A. cerana*, Wanju, Korea, 12. *A. cerana*, Hiroshima, Japan, 13. *A. cerana*, Kitahiroshima, Japan, 14. *A. cerana*, Kyoto, Japan, 15. *A. cerana*, Beijing, China, 16. *A. cerana*, Jiangxi, China, 17. *A. cerana*, Taiwan, China, 18. *A. cerana*, Yunnan, China, 19. *A. cerana*, Kathmandu, Nepal, 20. *A. cerana*, Vladivostok, Russia, 21. *A. mellifera*, Out group. * The Korean samples of *A. cerana* are indicated in bold.

Table S3. The ratio of non-synonymous to synonymous substitutions (dN/dS) between local populations of *A. cerana* and outgroup *A. mellifera* based on the exon sequences of the gene *VG* below the diagonal and its standard error (S.E.) above the diagonal

		**1**	**2**	**3**	**4**	**5**	**6**	**7**	**8**	**9**	**10**	**11**	12	13	14	15	16	17	18	19	20	21
		Standard error S.E.																				
**1***			**0.003**	**0.002**	**0.002**	**0.002**	**0.002**	**0.002**	**0.002**	**0.002**	**0.002**	**0.002**	0.002	0.003	0.003	0.003	0.003	0.004	0.002	0.003	0.003	0.010
**2**		**0.370**		**0.002**	**0.003**	**0.002**	**0.003**	**0.004**	**0.002**	**0.002**	**0.001**	**0.003**	0.003	0.003	0.003	0.004	0.004	0.004	0.003	0.003	0.004	0.010
**3**		**0.314**	**0.473**		**0.002**	**0.002**	**0.003**	**0.003**	**0.002**	**0.002**	**0.002**	**0.004**	0.002	0.002	0.002	0.003	0.003	0.004	0.003	0.003	0.004	0.010
**4**		**0.384**	**0.292**	**0.247**		**0.002**	**0.003**	**0.003**	**0.002**	**0.001**	**0.002**	**0.002**	0.002	0.002	0.003	0.003	0.003	0.004	0.002	0.003	0.004	0.010
**5**		**0.398**	**0.385**	**0.441**	**0.343**		**0.002**	**0.002**	**0.002**	**0.001**	**0.002**	**0.002**	0.002	0.002	0.002	0.003	0.003	0.004	0.002	0.003	0.003	0.010
**6**		**0.609**	**0.382**	**0.446**	**0.337**	**0.394**		**0.002**	**0.002**	**0.002**	**0.003**	**0.003**	0.002	0.003	0.003	0.004	0.004	0.004	0.003	0.004	0.002	0.010
**7**		**0.384**	**0.315**	**0.367**	**0.518**	**0.315**	**0.509**		**0.002**	**0.003**	**0.003**	**0.004**	0.003	0.003	0.004	0.004	0.004	0.004	0.003	0.004	0.003	0.010
**8**		**0.410**	**0.362**	**0.415**	**0.489**	**0.422**	**0.547**	**0.512**		**0.002**	**0.002**	**0.002**	0.002	0.003	0.003	0.003	0.003	0.004	0.002	0.003	0.002	0.010
**9**		**0.625**	**0.379**	**0.583**	**0.546**	**0.476**	**0.491**	**0.454**	**0.584**		**0.002**	**0.003**	0.002	0.002	0.002	0.003	0.003	0.002	0.002	0.003	0.003	0.010
**10**	dN/dS ratio	**0.474**	**0.695**	**0.821**	**0.422**	**0.517**	**0.382**	**0.385**	**0.482**	**0.502**		**0.003**	0.002	0.002	0.002	0.003	0.003	0.004	0.002	0.003	0.004	0.010
**11**		**0.408**	**0.433**	**0.409**	**0.829**	**0.299**	**0.328**	**0.158**	**0.488**	**0.614**	**0.439**		0.003	0.003	0.004	0.004	0.004	0.004	0.003	0.004	0.004	0.010
12		0.439	0.295	0.334	0.236	0.365	0.451	0.394	0.431	0.397	0.435	0.453		0.003	0.003	0.004	0.004	0.004	0.003	0.003	0.003	0.010
13		0.309	0.403	0.609	0.394	0.321	0.310	0.229	0.418	0.473	0.549	0.272	0.315		0.002	0.003	0.003	0.003	0.003	0.003	0.004	0.010
14		0.446	0.433	0.999	0.300	0.393	0.226	0.334	0.388	0.370	0.473	0.359	0.507	0.609		0.003	0.003	0.004	0.003	0.003	0.004	0.010
15		0.500	0.487	0.560	0.569	0.478	0.406	0.354	0.506	0.498	0.470	0.451	0.449	0.424	0.373		0.004	0.003	0.002	0.002	0.004	0.010
16		0.452	0.457	0.513	0.486	0.476	0.390	0.289	0.529	0.484	0.527	0.428	0.446	0.327	0.485	0.377		0.004	0.003	0.004	0.004	0.010
17		0.463	0.403	0.459	0.543	0.479	0.404	0.377	0.514	0.467	0.451	0.459	0.362	0.392	0.387	0.637	0.391		0.003	0.003	0.005	0.010
18		0.581	0.572	0.690	0.670	0.539	0.457	0.328	0.566	0.594	0.512	0.457	0.485	0.489	0.380	0.474	0.350	0.551		0.002	0.004	0.010
19		0.524	0.455	0.535	0.580	0.548	0.489	0.405	0.525	0.514	0.515	0.515	0.501	0.489	0.498	0.947	0.376	0.867	0.820		0.005	0.010
20		0.643	0.578	0.557	0.711	0.642	0.846	0.798	0.699	0.758	0.598	0.645	0.698	0.582	0.619	0.575	0.579	0.538	0.629	0.690		0.010
21		0.520	0.511	0.538	0.537	0.521	0.507	0.496	0.524	0.524	0.524	0.523	0.504	0.514	0.514	0.497	0.511	0.502	0.513	0.520	0.500	

Notes: 1. *A. cerana*, Gangneung, Korea, 2. *A. cerana*, Goseong, Korea, 3. *A. cerana*, Hongcheon, Korea, 4. *A. cerana*, Samcheok, Korea, 5. *A. cerana*, Sancheong, Korea, 6. *A. cerana*, Sangju, Korea, 7. *A. cerana*, Seoul, Korea, 8. *A. cerana*, Icheon, Korea, 9. *A. cerana*, Okcheon, Korea, 10. *A. cerana*, Uisung, Korea, 11. *A. cerana*, Wanju, Korea, 12. *A. cerana*, Hiroshima, Japan, 13. *A. cerana*, Kitahiroshima, Japan, 14. *A. cerana*, Kyoto, Japan, 15. *A. cerana*, Beijing, China, 16. *A. cerana*, Jiangxi, China, 17. *A. cerana*, Taiwan, China, 18. *A. cerana*, Yunnan, China, 19. *A. cerana*, Kathmandu, Nepal, 20. *A. cerana*, Vladivostok, Russia, 21. *A. mellifera*, Out group. *The Korean samples of *A. cerana* are indicated in bold.

Table S4. The Jukes-Cantor genetic distances between local populations of *A. cerana* and outgroup *A. mellifera* based on the exon sequences of the gene *VG* below the diagonal and its standard error (S.E.) above the diagonal

		**1**	**2**	**3**	**4**	**5**	**6**	**7**	**8**	**9**	**10**	**11**	12	13	14	15	16	17	18	19	20	21
		Standard error S.E.																				
**1***	Jukes-Cantor genetic distances		**0.001**	**0.001**	**0.001**	**0.001**	**0.001**	**0.001**	**0.001**	**0.001**	**0.001**	**0.001**	0.001	0.001	0.001	0.001	0.001	0.001	0.001	0.001	0.001	0.004
**2**		**0.004**		**0.001**	**0.001**	**0.001**	**0.001**	**0.001**	**0.001**	**0.001**	**0.001**	**0.001**	0.001	0.001	0.001	0.001	0.002	0.001	0.001	0.001	0.001	0.001
**3**		**0.004**	**0.002**		**0.001**	**0.001**	**0.001**	**0.001**	**0.001**	**0.001**	**0.001**	**0.001**	0.001	0.001	0.001	0.001	0.002	0.001	0.001	0.001	0.002	0.005
**4**		**0.003**	**0.003**	**0.003**		**0.001**	**0.001**	**0.001**	**0.001**	**0.001**	**0.001**	**0.001**	0.001	0.001	0.001	0.001	0.001	0.001	0.001	0.001	0.001	0.004
**5**		**0.003**	**0.003**	**0.004**	**0.003**		**0.001**	**0.001**	**0.001**	**0.001**	**0.001**	**0.001**	0.001	0.001	0.001	0.001	0.001	0.001	0.001	0.001	0.001	0.004
**6**		**0.004**	**0.005**	**0.005**	**0.004**	**0.004**		**0.001**	**0.001**	**0.001**	**0.001**	**0.001**	0.001	0.001	0.001	0.001	0.001	0.001	0.001	0.001	0.001	0.004
**7**		**0.004**	**0.006**	**0.006**	**0.005**	**0.004**	**0.004**		**0.001**	**0.001**	**0.001**	**0.001**	0.001	0.001	0.001	0.001	0.001	0.001	0.001	0.001	0.001	0.005
**8**		**0.004**	**0.004**	**0.004**	**0.004**	**0.004**	**0.005**	**0.005**		**0.001**	**0.001**	**0.001**	0.001	0.001	0.001	0.001	0.001	0.001	0.001	0.001	0.002	0.005
**9**		**0.005**	**0.003**	**0.003**	**0.003**	**0.003**	**0.004**	**0.005**	**0.005**		**0.001**	**0.001**	0.001	0.001	0.001	0.001	0.001	0.001	0.001	0.001	0.002	0.004
**10**		**0.005**	**0.003**	**0.003**	**0.005**	**0.004**	**0.005**	**0.007**	**0.005**	**0.004**		**0.001**	0.001	0.001	0.001	0.001	0.001	0.001	0.001	0.001	0.002	0.005
**11**		**0.003**	**0.005**	**0.006**	**0.004**	**0.003**	**0.004**	**0.003**	**0.005**	**0.005**	**0.006**		0.001	0.001	0.002	0.001	0.002	0.001	0.001	0.001	0.001	0.004
12		0.004	0.004	0.004	0.004	0.004	0.005	0.006	0.005	0.005	0.005	0.005		0.001	0.001	0.002	0.002	0.001	0.001	0.001	0.001	0.005
13		0.005	0.005	0.005	0.005	0.005	0.006	0.005	0.007	0.005	0.006	0.005	0.006		0.001	0.001	0.001	0.001	0.001	0.001	0.001	0.005
14		0.006	0.005	0.004	0.006	0.005	0.005	0.009	0.006	0.004	0.004	0.008	0.006	0.006		0.001	0.001	0.001	0.001	0.001	0.002	0.005
15		0.010	0.009	0.010	0.009	0.009	0.009	0.009	0.010	0.008	0.009	0.009	0.010	0.010	0.010		0.001	0.001	0.001	0.001	0.002	0.004
16		0.007	0.007	0.007	0.006	0.006	0.007	0.006	0.008	0.006	0.007	0.007	0.008	0.006	0.007	0.009		0.002	0.001	0.002	0.002	0.004
17		0.009	0.008	0.008	0.007	0.008	0.009	0.008	0.010	0.007	0.009	0.008	0.009	0.008	0.010	0.009	0.009		0.001	0.001	0.002	0.005
18		0.007	0.006	0.007	0.006	0.006	0.007	0.006	0.008	0.005	0.007	0.007	0.008	0.007	0.007	0.007	0.007	0.007		0.001	0.002	0.004
19		0.007	0.005	0.007	0.007	0.007	0.008	0.008	0.008	0.006	0.007	0.007	0.007	0.007	0.008	0.009	0.008	0.007	0.007		0.002	0.005
20		0.009	0.012	0.011	0.010	0.010	0.009	0.008	0.011	0.011	0.012	0.008	0.010	0.010	0.013	0.015	0.012	0.014	0.012	0.013		0.005
21		0.067	0.066	0.066	0.065	0.066	0.067	0.066	0.068	0.065	0.067	0.066	0.068	0.066	0.067	0.068	0.065	0.067	0.065	0.068	0.070	

Notes: 1. *A. cerana*, Gangneung, Korea, 2. *A. cerana*, Goseong, Korea, 3. *A. cerana*, Hongcheon, Korea, 4. *A. cerana*, Samcheok, Korea, 5. *A. cerana*, Sancheong, Korea, 6. *A. cerana*, Sangju, Korea, 7. *A. cerana*, Seoul, Korea, 8. *A. cerana*, Icheon, Korea, 9. *A. cerana*, Okcheon, Korea, 10. *A. cerana*, Uisung, Korea, 11. *A. cerana*, Wanju, Korea, 12. *A. cerana*, Hiroshima, Japan, 13. *A. cerana*, Kitahiroshima, Japan, 14. *A. cerana*, Kyoto, Japan, 15. *A. cerana*, Beijing, China, 16. *A. cerana*, Jiangxi, China, 17. *A. cerana*, Taiwan, China, 18. *A. cerana*, Yunnan, China, 19. *A. cerana*, Kathmandu, Nepal, 20. *A. cerana*, Vladivostok, Russia, 21. *A. mellifera*, Out group. * The Korean samples of *A. cerana* are indicated in bold.

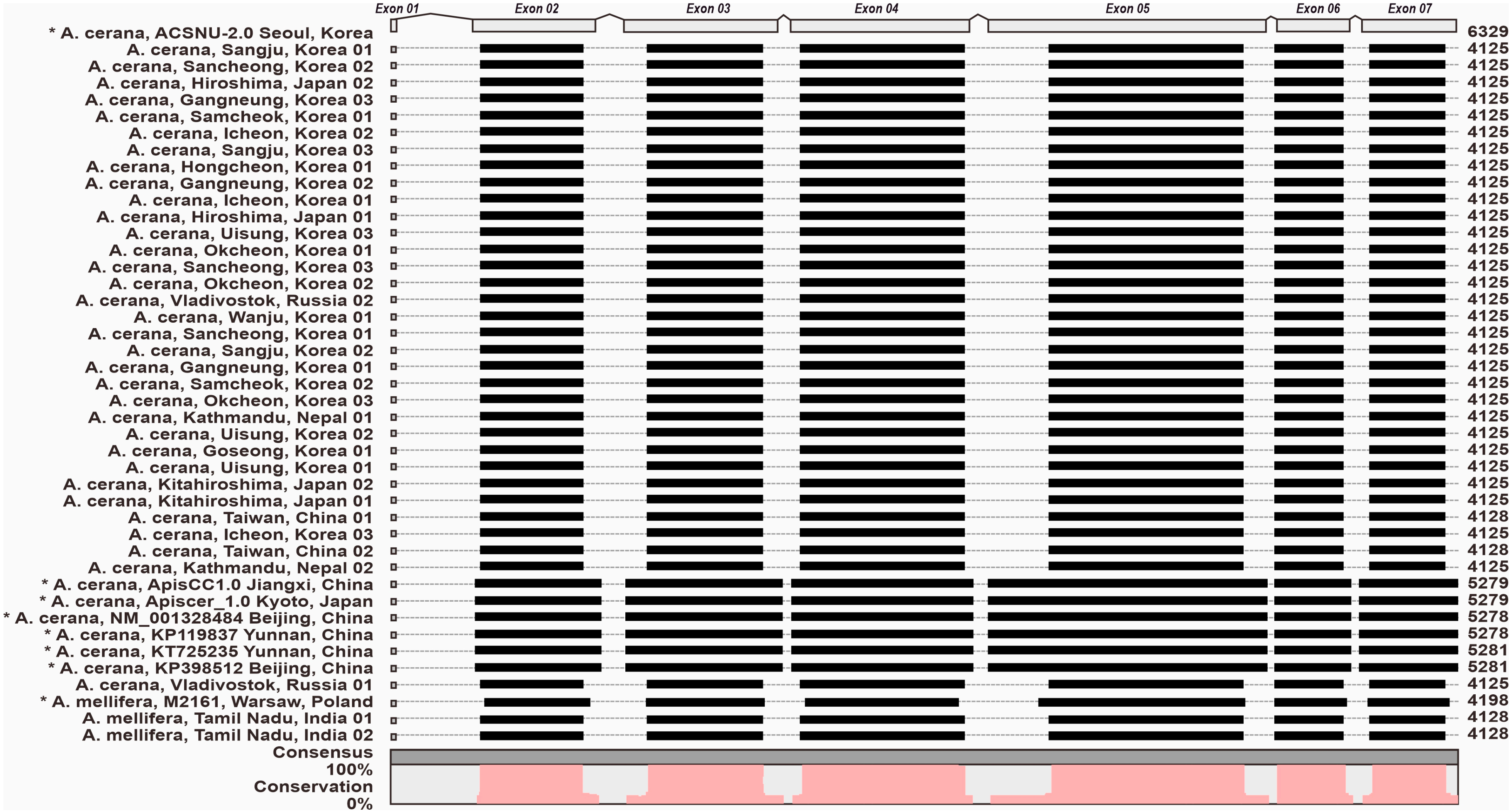

Figure S1. The aligned exons of the gene *VG* of *A. cerana* and outgroup *A. mellifera* samples.
